# The PMC Turbo Balloon Mission to Measure Gravity Waves and Turbulence in Polar Mesospheric Clouds: Camera, Telemetry, and Software Performance

**DOI:** 10.1029/2020ea001238

**Published:** 2020-08-02

**Authors:** Carl Bjorn Kjellstrand, Glenn Jones, Christopher Geach, Bifford P. Williams, David C. Fritts, Amber Miller, Shaul Hanany, Michele Limon, Jason Reimuller

**Affiliations:** 1 Department of Physics, Columbia University, New York, NY, USA; 2 Rigetti Computing, Berkeley, CA, USA; 3 School of Physics and Astronomy, University of Minnesota, Minneapolis, MN, USA; 4 Boulder Division, GATS, Boulder, CO, USA; 5 Department of Physics and Astronomy, University of Southern California, Los Angeles, CA, USA; 6 Department of Physics and Astronomy, University of Pennsylvania, Philadelphia, PA, USA; 7 Integrated Spaceflight Services, Boulder, CO, USA

## Abstract

The Polar Mesospheric Cloud Turbulence (PMC Turbo) instrument consists of a balloon-borne platform which hosts seven cameras and a Rayleigh lidar. During a 6-day flight in July 2018, the cameras captured images of Polar Mesospheric Clouds (PMCs) with a sensitivity to spatial scales from ~20 m to 100 km at a ~2-s cadence and a full field of view (FOV) of hundreds of kilometers. We developed software optimized for imaging of PMCs, controlling multiple independent cameras, compressing and storing images, and for choosing telemetry communication channels. We give an overview of the PMC Turbo design focusing on the flight software and telemetry functions. We describe the performance of the system during its first flight in July 2018.

## 1. Introduction

Gravity waves (GWs) and the instabilities and turbulence associated with GW breaking play an important role in the dynamics and structure of the atmosphere at local and global scales. They are thought to contribute the major vertical transports of energy and momentum from tropospheric sources to higher altitudes (Fritts & Alexander, 2003). Specific events can be compared to models of turbulence and can help evaluate how closely models match nature.

Polar Mesospheric Cloud (PMCs) form near the local minimum atmospheric temperature at the polar summer mesopause where trace water vapor becomes supersaturated and allows heterogeneous nucleation on meteoric smoke particles. The temperature minimum occurs during the polar summer due to adiabatic cooling by the mean circulation upward and equatorward driven, which is driven by GW dissipation and momentum deposition. GW and turbulence dynamics have large spatial scales in the PMC region (~83 km) relative to lower altitudes. Bright PMCs are few tens to few hundred meters thick and are thus sensitive tracers of small-scale dynamics (Baumgarten & Fritts, 2014; Taylor et al., 2011; Witt, 1962).

GWs are not unique to the mesosphere and lower thermosphere (MLT) region of the atmosphere. Turbulence dynamics associated with GW breaking are also present in lower regions of the atmosphere and within oceans and large lakes. However, the small scale of such dynamics and the absence of a tracer of appropriate size impose challenges on the observation of such dynamics in other mediums. Observing PMCs in the MLT is not without challenges, but the use of a balloon-borne observational platform allows higher resolution, more frequent, and higher signal-to-noise observations than from the ground. Stratospheric balloons fly at half the distance to PMCs compared to ground observatories doubling the resolution for the same hardware and viewing angles. Lower tropospheric clouds often block the line of sight (LOS). Since PMCs occur over the summer poles, they are visible from the ground when the PMC layer is sunlit and the ground is dark. This creates a narrow range of latitudes and short season where observation with a good signal-to-noise ratio (SNR) is possible. Current space-based observational platforms such as the Cloud Imaging and Particle Size (CIPS) instrument (McClintock et al., 2009) have much lower spatial resolution of kilometers per pixel.

The scientists working on the E and B Experiment (EBEX), a balloon-borne cosmology mission, discovered the utility of balloon-borne platforms in observing PMCs (Miller et al., 2015). In the Antarctic summer of 2012–2013, the experimental cosmology payload EBEX launched from McMurdo Station, Antarctica with the objective of measuring polarization modes of the Cosmic Microwave Background (described in The EBEX Collaboration et al., 2018; The EBEX Collaboration, Aboobaker, Ade, Araujo, Aubin, Baccigalupi, Bao, Chapman, Didier, Dobbs, Geach, et al., 2018; The EBEX Collaboration, Aboobaker, Ade, Araujo, Aubin, Baccigalupi, Bao, Chapman, Didier, Dobbs, Grainger, et al., 2018). The payload included star cameras to find the pointing of the main telescope, which serendipitously imaged PMCs with unprecedented signal to noise and spatial resolution. The data collected by the EBEX star cameras produced valuable insight into dynamics in the MLT (Fritts et al., 2017; Miller et al., 2015). We designed PMC Turbo to take advantage of the instrumental heritage of EBEX while increasing the field of view (FOV) and imaging cadence to capture both large- and small-scale turbulence dynamics in the MLT.

PMC Turbo payload includes optical cameras which capture images of PMCs at a spatial resolution comparable to the highest achieved to date (Baumgarten & Fritts, 2014) and with the most rapid cadence, as well as a Rayleigh lidar which is described in Kaifler et al. (2020). Our science goals for PMC Turbo were to identify the dominant GW, instability, and turbulence dynamics that define the character and scales of GW dissipation events and to assess the magnitudes and scales of GW momentum fluxes and understand their implications for MLT forcing and secondary GW radiation. To this end, we selected camera hardware to attain spatial resolution approaching the inner scale of turbulence (~20 m) and captured images at a ~2-s cadence to capture GW dynamics evolving on timescales of minutes. The high cadence and resulting large volume of images that were expected during flight required the development of specialized camera control, image acquisition, and telemetry software. These requirements are unique compared to other balloon payloads (see, e.g., Galitzki et al., 2014; The EBEX Collaboration, Aboobaker, Ade, Araujo, Aubin, Baccigalupi, Bao, Chapman, Didier, Dobbs, Grainger, et al., 2018) that routinely implement *star* cameras, which aid in real-time attitude control and post-flight attitude reconstruction. PMC Turbo flew over the Arctic in July 2018 and captured some 6 million images. We have published our initial science findings in an overview paper (Fritts et al., 2019), and analysis of individual events is currently in progress. In this paper, we describe the camera and control hardware, as well as the control and image acquisition software, which were necessary to fulfill the science goals.

## 2. PMC Turbo Instrumentation

### 2.1. Instrument Overview

**Figure 1.**
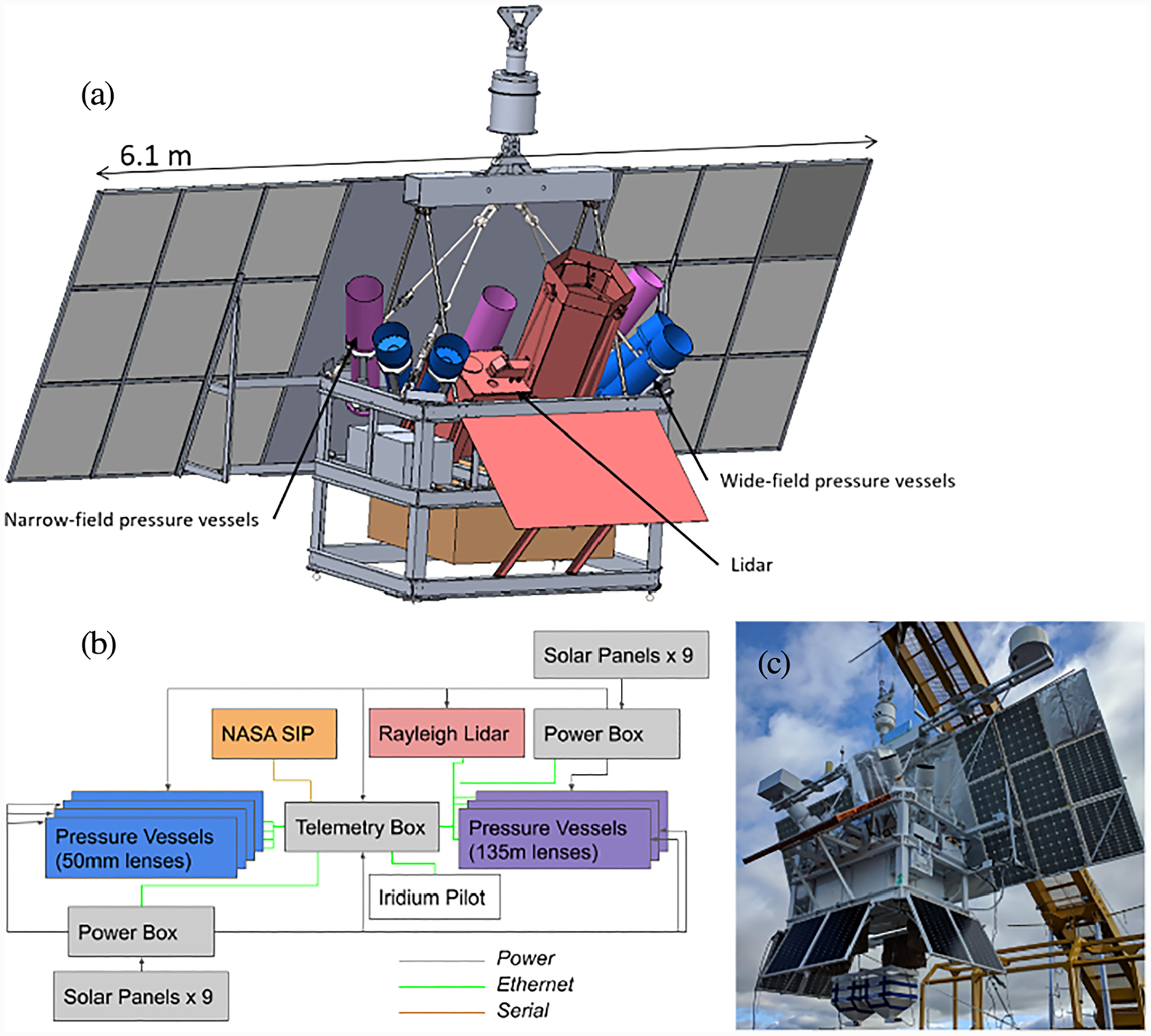
Panel a: Sketch of the PMC Turbo payload. There are three narrow-field cameras in pressure vessels (purple), four wide-field cameras in pressure vessels (blue), the NASA support instrumentation package (SIP) (orange), and a lidar and associated telescope and radiator (red). Panel b: Flowchart of the power and network connections of the subsystems of PMC Turbo. Panel c: Photo of the integrated PMC Turbo gondola hanging from the launch vehicle shortly before launch.

The PMC Turbo gondola consists of an aluminum frame supporting seven camera pressure vessels, one Rayleigh lidar pressure vessel and telescope, two infrasound piggyback instruments, a telemetry system, and a power system. Each of the seven pressure vessels contains a lens, a camera, four spinning hard disks, and a computer performing data acquisition and flight control. Four of the pressure vessels have cameras with wide-field lenses mounted. These cameras are highlighted in blue in Figure 1a. Together these cameras cover an FOV of 100° × 40°. The other three cameras, highlighted in purple in Figure 1a, have narrow-field lenses mounted. Each of the narrow FOVs covers 10° × 15° pointed at the overlap of two wide FOVs. The lidar, marked in orange, is the first high-powered lidar to be successfully flown aboard a balloon-borne platform and will be described in a dedicated paper. Figure 1c shows a photo of the integrated gondola.

**Figure 2.**
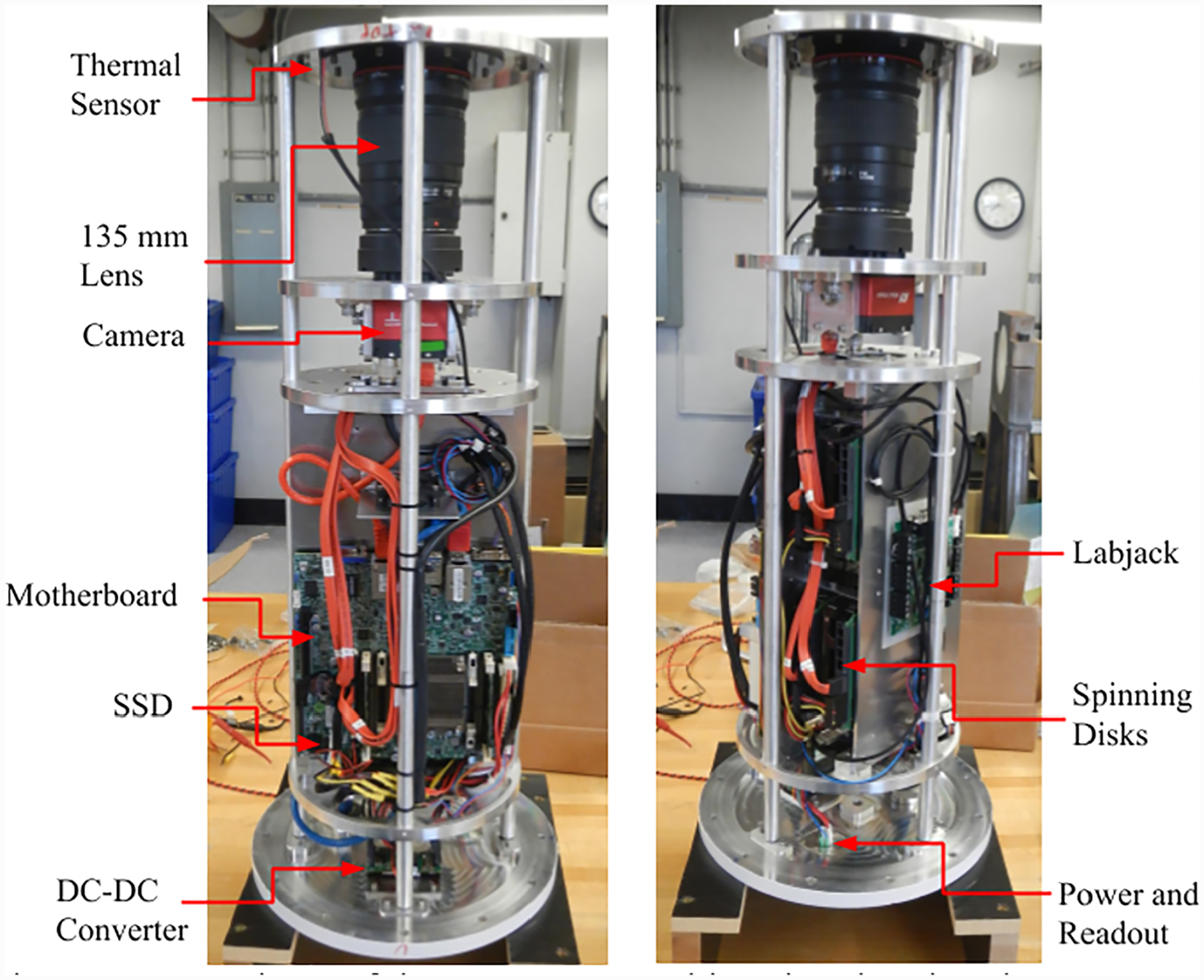
Two views of the pressure vessel interior showing the camera, computer, disks, and sensor system.

We used Allied Vision Prosilica GT 4907 cameras, which contain Kodak 16070 charge-coupled device (CCD) image sensors. These CCDs have 3,232 × 4,864 pixels with a well depth of 40,000 electrons and a quantum efficiency of 20–35% in the probed wavelengths. We mounted a Hoya #25A filter in front of each lens to improve the SNR of features traced in PMCs by blocking wavelengths shorter than 600 nm, and we image from 600 nm to the near infrared. The filter suppresses the prevalent blue-scattered light from the residual atmosphere, while attenuating the white light from PMCs less than the background sky brightness. Combining the wide FOV 50 mm F/1.24 lens with the 7.4-micron pixel size, the camera captures 40° × 27° FOV with a resolution of 30.5 arcseconds/pixel. When projected from the gondola at 38-km altitude to the PMC layer at 83 km, the image has 8 m/pixel of resolution at the top of the image (25° off zenith). The narrow FOV 135 mm F/2 lens mounted on the same camera has 10° × 15° FOV with 11.3 arcseconds/pixel. At the PMC layer, this corresponds to 3 m/pixel. Figure 2 shows the arrangement of the cameras, lenses, sensors, and computer hardware of the pressure vessels.

The cameras include a lens mount which controls the lens’s focus and aperture. We control each camera through a software interface, and we communicate with the cameras via an Ethernet cable, using a standard GigE Vision link, which connects the camera to the pressure vessel computer. The flight control software controls the aperture, focus, and exposure through this interface. During flight, we fine-tuned focus, aperture, and exposure to adapt to live sky conditions. We bolted simple baffles to the lens side of the pressure vessels. We used aluminum tubes of the same diameter as the pressure vessels painted white on the exterior and black on the interior. The baffles are visible on the lens side of the pressure vessels in Figures 1a and 1c.

Each pressure vessel contains a computer running the image capture process. Any of the computers can also manage the flight control system for redundancy, but only one was assigned to do so during flight. The motherboards are Supermicro mini ITX A1SAI-2750F-O server boards. We selected this motherboard because it provided a small form factor, ample processing power, low-power consumption, error-correcting code (ECC) memory, support for enough hard disks, and Ethernet ports, all at a small fraction of the cost of an industrial or ruggedized computer more typically used for balloon applications. The motherboards include an industry standard Intelligent Platform Management Interface (IPMI), which we used to set up the system and diagnose issues over the Ethernet interface without needing to reopen the sealed pressure vessels. In each pressure vessel, four Western Digital Red 8TB (WD80EFAX) spinning disks store the experiment data. The computer writes images to the four disks in a roughly alternating manner so that a disk failure does not remove a continuous section of flight. We also wrote one image every few minutes to a generic 128-GB SanDisk Cruzer USB flash drive as an alternative storage medium, in case all spinning disks failed unexpectedly. We set up the Debian operating system on a RAID array with three times redundancy on two spinning disks and a dedicated ADATA ISMS312 industrial 32-GB SSD.

DC power is supplied to the pressure vessel directly from the battery bus, and a high-efficiency DC/DC converter (UIE48T10120) regulates to +12 VDC with and directly powers the motherboard. All other components inside the pressure vessel are powered from the motherboard, eliminating the need for additional relays or control circuitry. A LabJack U3-HV USB data acquisition unit records the temperature from several Analog Devices AD590 sensors, and NXP MPX4250A board mount sensor measures the internal pressure.

### 2.2. Camera Comparison

PMC Turbo narrow-field cameras capture images with very similar resolution to the EBEX star cameras. Our wide-field cameras record images with a similar resolution to ground-based cameras. The innovation of PMC Turbo comes from the change in latitude and altitude that allows for observation of more polar PMCs throughout the day. PMC brightness and prevalence increase toward the pole, and ground-based experiments cannot observe PMCs during the long days of polar summers. Even accounting for a flight of a few days, these factors allow PMC Turbo to collect many more PMC images in a season than ground-based cameras. Compared to the CIPS satellite PMC images, PMC Turbo probes vastly different scales; a single pixel of CIPS records 1 km × 2 km on the PMC plane, and the satellite observes the entire pole over the course of a day. A few days before we launched PMC Turbo, the Stratospheric Observations of Noctilucent Clouds (SONC) instrument launched from the Moscow region and flew for 1.7 hr, reaching an altitude of 20.4 km (Dalin et al., 2020). SONC hosted an optical camera with a wide-field lens (FOV of 110° × 82°) and a resolution varying from ~30 to ~3,000 m depending on the elevation angle. Taken together, our wide-field cameras covered a FOV of roughly 100° × 40°, with a resolution varying from ~9 m at large elevation angles to ~45 m at the lowest elevation angles. Note that motion blur reduced our effective resolution (described in detail in the following section), so we generally attained an effective resolution around 20 m, similar to the inner scale of turbulence Table 1.

### 2.3. Exposure Time Considerations

The range of exposure times is constrained by the competing factors of wanting to integrate for as long as possible to maximize SNR while also avoiding excessive blurring caused by the relative motion of the PMC field on the image sensor. This “motion blur” is a result of the motion of the camera platform and bulk advection of the clouds themselves. A NASA-supplied rotator controlled the orientation of the payload. It kept the gondola pointed in azimuth anti-Sun direction to optimally illuminate the solar cells and to keep sunlight out of the cameras and lidar. The NASA rotator technicians predicted azimuth oscillations with amplitude of 1° and period of 42 s. In reality, we observed an amplitude of an eighth of a degree with a period of ~50 s, which contributed negligible motion blur.

Unexpectedly, we observed an oscillation in our images with a period of ~10 s. The oscillation varied in direction on timescales of minutes. The oscillation is the largest contributor to our motion blur, as it has an amplitude of <0.1° or ~110 m when the images are projected onto the PMC plane. With a 10-s period, this motion introduces 70 m/s of worst case apparent motion with a root mean square of 50 m/s. We examined the projected images and found that not only did the oscillation vary in direction but it also varied in frequency over time. We have not conclusively identified the source of the short timescale jitter. However, we have no reason to believe that our flight train’s mechanical attributes changed for any reason other than temperature, and we do not find a relationship between the jitter frequency and temperature. There is atmospheric turbulence at the altitude of the gondola, and we find it plausible that the winds buffeted the gondola around or had a shear between the gondola and balloon. While we find the study of turbulence at the gondola altitude intriguing, we did not design the experiment as a sensor of turbulence at 38 km. We plan to include further sensors on future missions to measure turbulence at the gondola altitude.

We predicted 50 ± 20 m/s of bulk velocity of the PMCs due to background winds. The bulk velocity we observe by tracking features varied throughout flight but fell within the predicted range. The high-frequency oscillation varied in direction on short timescales and had a period several times longer than our image capture cadence. The effect of the motion blur on our image resolution therefore varies throughout individual events. The inner scale of turbulence at the PMC layer is about 20 m. Depending on the exposure settings used, the motion blur affected scales similar to the inner scale of turbulence. This has imposed challenges in measuring the power spectrum of turbulence in the PMC layer, but our larger scale dynamics analysis tends to study features with scales of hundreds to thousands of meters, so the motion blur did not prevent us from achieving our primary science goals. In these analyses, SNR is more useful than spatial resolution, so we bin adjacent pixels, reducing the effect of recorded motion blur.

Lower exposure times prevent blurring in our images. However, lower exposure times also reduce the SNR of imaged objects. During flight, we alternated the exposure mode between auto-exposures and static exposure lengths due to varying sky conditions. In the auto-exposure mode, we used an auto-exposure algorithm which automatically and periodically evaluated the statistics of a recently captured image and then adjusted the exposure to the CCD well depth to within an adjustable range. We adjusted the trigger interval and frame rate to match this exposure. The auto-exposure mode attempts to optimize the SNR for a single frame but introduces more motion blur relative to shorter exposure times. We generally set narrow-field cameras to longer exposure durations, since the narrow-field lenses had lower SNR due to the narrower apertures. In general, the auto-exposure algorithm found an optimal exposure of 200–300 ms for wide-field cameras. We wanted to test both the auto-exposure and manual exposure methods during flight. Auto-exposure gave the best S/N of the sky without overexposing, so by default, we left the cameras in auto-exposure mode. After we confirmed the presence of PMCs in our images for several hours, we switched to shorter manual exposures, so we could analyze the utility of both modes with PMC images. This method prioritized auto-exposure, and by 12 July, we knew that we had captured many PMC images. To ensure we captured sufficient manual exposures to analyze, we manually set the exposure of the wide-field cameras to 100 ms for the duration of 12 July. We observed light reflections into one narrow-field camera during flight. Auto-exposure did not handle this situation well, so we used the flexibility of our exposure control to manually set exposure for that camera and to avoid losing SNR in the entire image by allowing the section of the image with the reflection to over-expose. In the last days of flight, we set the narrow-field cameras to a short exposure time to test burst-mode capabilities for future flights.

### 2.4. Image Cadence Capabilities

An image recorded by the camera’s CCDs is temporarily stored to a buffer on the camera. The camera computer retrieves the image via the Ethernet interface. The pipeline software running on the camera computer receives the image and writes the data to one of the four data disks situated in the same pressure vessel. The hardware of the camera allows for a capture rate of nearly 8 frames per second (fps) into its internal buffer, and we used this functionality to capture bursts of images at low-exposure times. However, the gigabit Ethernet only allows the computer to retrieve 3.5 fps. For extended flights, total available disk space also limits the number of images that can be retrieved, although the Arctic flight did not approach that limit. Finally, image exposure necessarily limits frame rate; for example, we cannot capture more than 4 fps with 250-ms exposures. In practice, we did not approach the frame rate limits of PMC Turbo. We typically captured bursts of four 250-ms exposures, separated by the hardware limit of 60 ms between each frame. We set the bursts to capture every 2 seconds. We did not have a pressing science purpose to capture images at a higher cadence.

Stitching together images from each of the seven cameras into one large FOV is straightforward if all of the exposures are synchronized. We were able to obtain submillisecond exposure synchronization by using the precision time protocol (PTP) supported by the cameras to keep the cameras’ internal timestamp aligned with each computer and using network time protocol (NTP) to synchronize all of the computers to each other and to UTC.

Higher image cadence reduces the effects of motion blur and enables the option of coadding to increase S/N. The images are captured in bursts synchronized across the cameras. The computer sends a command to arm the camera, and the camera starts a burst at the next whole second. It exposes the CCD for the set time (with a minimum exposure time of 35 ms), then waits a specified time, and exposes again for the same time.

### 2.5. PMC Turbo Performance During Flight

**Figure 3.**
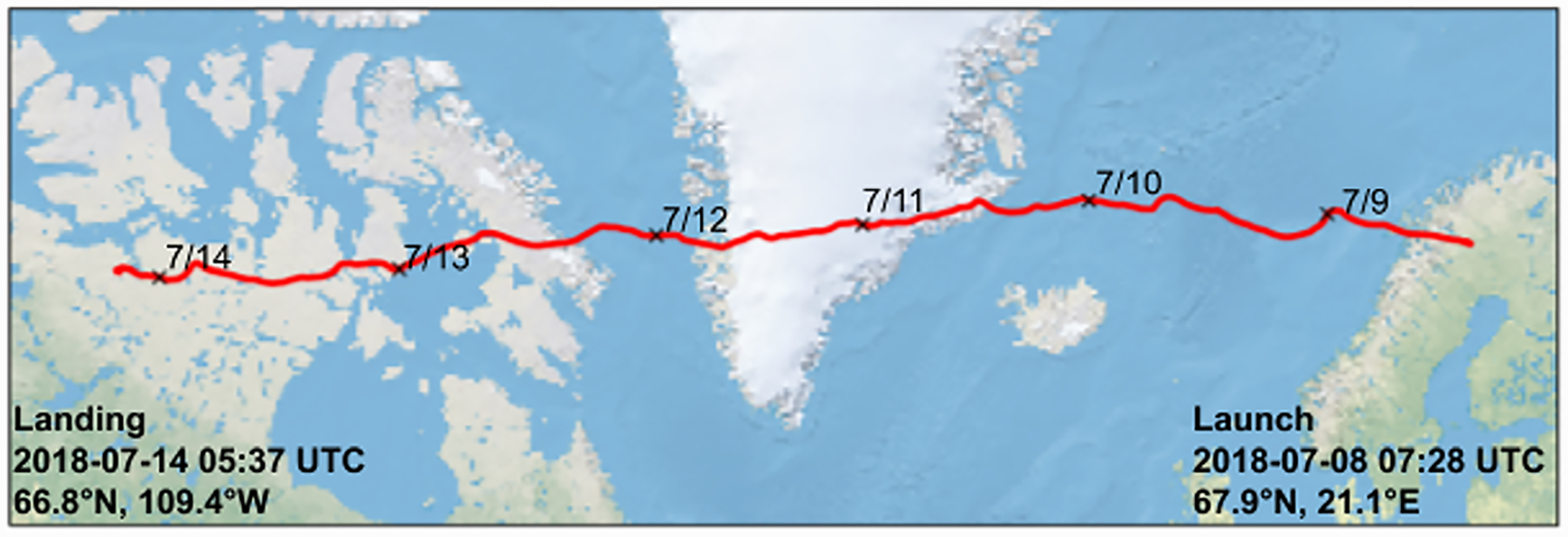
The flight path of the PMC Turbo gondola from launch to landing.

We launched PMC Turbo from the Esrange Space Center in northern Sweden on 7 July 2018 (07:30 UTC, 21.1°E, 67.9°N). The balloon floated westward over Greenland and Baffin Island before termination over western Nunavut on 14 July (05:30 UTC, 109.4°E, 66.8°N; see Figure 3). The balloon remained between 37- and 39-km altitude between ascent and termination. Upon landing, the instrument sustained only superficial damage and was recovered by 22 July. During flight, the PMC Turbo cameras captured 6 million images, of which 60% contain images of PMCs. The lidar also operated nominally and successfully measured PMC backscatter and middle atmospheric temperature throughout the flight. Internal temperatures of the pressure vessels varied from −10°C to +35°C during flight and down to −20°C during ascent.

## 3. Software

**Figure 4.**
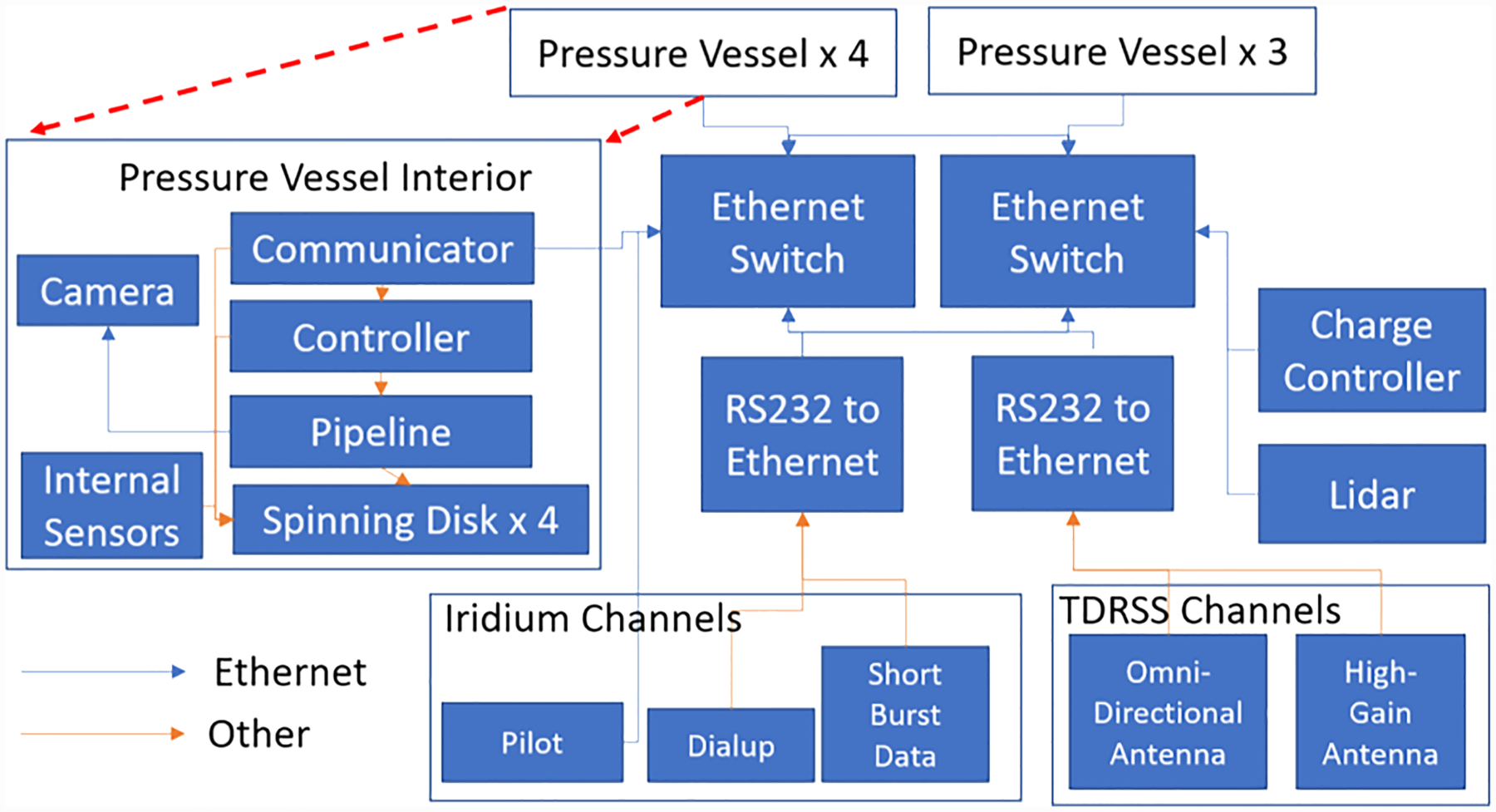
Overview of software and payload software architecture and network.

Most of the functionality of the flight software occurs in three processes which are named the *pipeline*, the *communicator*, and the *controller*. Figure 4 shows a simplified visual overview of the software and hardware network. The software discussed in this section will be distributed to the community in early 2021 via the PyHC Skywinder package.

### 3.1. The Pipeline

The *pipeline* is responsible for interfacing with the camera. It sends commands to the camera, arms the camera to capture an image, receives the image data, and writes the image to one of the four data disks. The *pipeline* performs lossless compression on the images when it stores them on the spinning disks using the blosc library (The Blosc Developers, 2020). This compression results in roughly 20 MB per image file. The CCDs we used have a pixel depth of 12 bits, and we primarily used lossless compression to avoid packing and unpacking 12-bit data. In the absence of any external commands, the *pipeline* commands the camera to capture images at a regular interval and stores those images to disk. The *pipeline* starts this process automatically at boot-up.

### 3.2. The Communicator

The *communicator* coordinates between the pressure vessels and communicates with ground operators. It aggregates housekeeping data, provides status reports and receives, relays, and responds to commands. Each camera computer runs a *communicator*, and the ground operators designate one *communicator* as the leader. The leader can be manually assigned from among the computers via a ground-command addressed to all cameras, but it was not necessary to switch leaders during flight since the default leader never failed. The *communicator* which has been assigned to be the leader listens to received ground-commands and responds appropriately including relaying command verification and providing housekeeping and image data. While each camera computer captures images for its own pressure vessel and keeps track of its own housekeeping, it does not send data on the network unless the leader *communicator* requests it. The leader also aggregates data from the lidar and charge controller of the power system.

### 3.3. The Controller

The *controller* interprets commands from the *communicator* and relays them to the *pipeline* in order to control camera settings and retrieves images from the data disks. During a focus sweep, the camera takes images at a range of focus steps and downlinks the images for ground review. The *controller* interprets the focus sweep command from the communicator and automatically prepares and relays commands to adjust focus step settings and retrieve images taken at each focus step. The *controller* also retrieves specific images and image sets requested by the communicator. In the absence of requests sent from the ground, the *controller* grabs the latest image, downsamples it, and applies lossy JPEG compression in preparation for downlinking according to the last specified parameters. The *controller* can also grab raw files (such as a raw housekeeping log or image) for downlink. For example, a ground-based user could request the default downlinked image to be a 512 × 512 pixel section selected from any location in the image at any possible downsample resolution (a resolution reachable via integer division of the original resolution of the image). In the absence of other commands, the *controller* passes the processed image to the communicator when the communicator requests the latest image.

### 3.4. Image Processing

**Figure 5.**
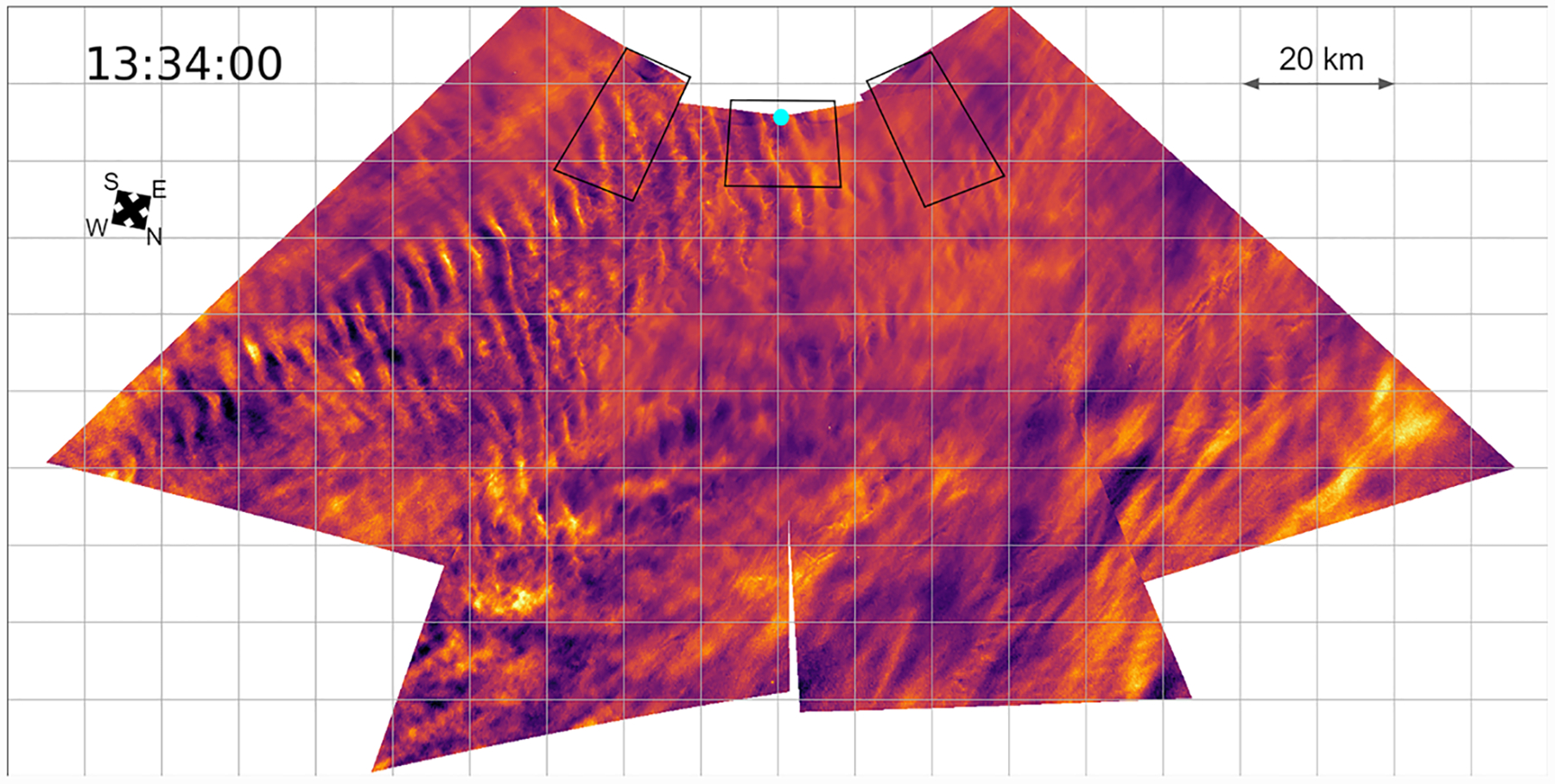
Four wide-field images processed and projected using pointing solutions with the approximate fields of view of the narrow-field cameras (black frames) and the approximate position of the lidar beam (cyan dot). The left side of the projected image features Kelvin-Helmholtz instabilities with a wavelength of ~5 km. We are currently analyzing this event.

We analyze images after implementing a standard flat-fielding procedure and normalizing for equal exposure time. All images are recorded in monochrome, but we typically apply false coloring to enhance visible contrast. The effect of the flat-fielding can be seen by comparing the first two images in Figure 5.

**Figure 6.**
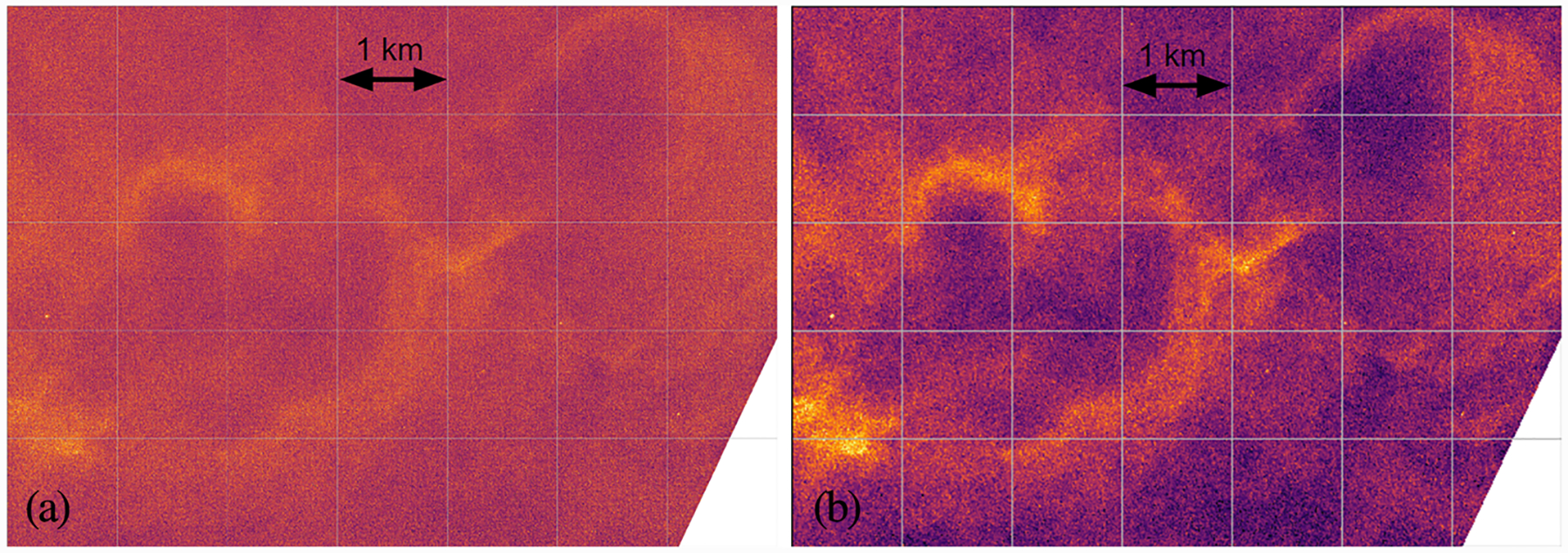
Medium-scale vortex rings captured by one of our narrow-field cameras. Panel a shows the image data with no pixel binning projected onto the PMC plane with a resolution of 4 m/projected pixel. We generally bin our pixels to increase our signal-to-noise ratio when the full resolution is not required to resolve dynamics of interest. Panel b shows the image with 4 × 4 pixel binning and projected onto the PMC plane with a resolution of 12.5 m/projected pixel.

The cameras capture images on a rectangular CCD, with cameras pointed at various angles. This introduces rectilinear distortion, which we remove by finding the pointing of the images using background star fields, and mapping each pixel to the proper location on the sky. We find the pointing of each camera by locating bright stars and inputting the star field into the Astrometry.net software package which identifies the stars based on their relative locations and then calculates right ascension and declination of every pixel (Lang et al., 2010). We convert these coordinates to altitude and azimuth using the GPS logs from flight. We choose a Cartesian grid on the PMC plane, map each location on the Cartesian grid to a pixel coordinate of an image, and assign the pixel value of the image at that pixel coordinate. With sufficiently high resolution on our Cartesian grid, we lose no information while projecting the source image. We demonstrate the projection and removal of spatial distortion of an image in Figure 5, and we show the projection of coincident flat-fielded images from the four wide-field cameras in Figure 6.

### 3.5. Telemetry

We downlink data during flight to monitor payload performance, to adjust the camera parameters to account for different sky conditions, and to insure the team against an unrecoverable payload. Balloon-borne payloads occasionally break upon landing or land in unrecoverable locations. The payload includes four communications antennas: two communicate with the Iridium network, and two with the TDRSS network. TDRSS consists of a network of communication satellites that have partial coverage of the polar regions due to their equatorial orbit. The Iridium network consists of satellites in low-Earth orbit and allows for better reception at the poles. We access the Iridium network over three links: Iridium Pilot, the short-burst data, and the dialup mode. Pilot has a separate dedicated antenna, an order of magnitude larger bandwidth, and uses a different communication protocol from other Iridium channels. Whenever possible, we used Iridium Pilot, since the network consistently reaches a bandwidth of 100 kilobits per second (kbps). The Iridium dialup mode has ~2-kbps bandwidth compared to the ~100 kbps of Pilot but provides an alternate channel on a separate antenna. The short-burst data mode reliably sends a 255-byte message at a 1-min cadence. We used these short messages to send essential housekeeping data. The TDRSS system includes a high-gain antenna and an omnidirectional antenna. The high-gain channel has a bandwidth of around 92 kbps, while the omnidirectional antenna gives 6 kbps. The gondola also communicated with a LOS system for the first ~24 hr of flight.

We communicated with the telemetry hardware via NASA hardware through an RS-232 interface. We used two RS-232 to Ethernet converters to connect to this interface and wrote communication software capable of communicating over a variety of links. We connected to Iridium Pilot directly via Ethernet. The consolidation of our network over Ethernet and standardization of communications allowed us to implement a distributed flight control system. As described in section 3.2, any communicator could assume leadership responsibilities, and the single network simplifies status monitoring. The consolidated Ethernet network also enabled a highly redundant network and guarded against single-point failures. The two RS-232 Ethernet converters and direct Ethernet connection to Iridium Pilot ensure that no single point of failure removes communication to all the channels. Our telemetry box includes two Ethernet switches connected to each other. Half of the pressure vessels, one of the two power boxes, and one RS232-Ethernet converter connected to each switch. This ensured that if either Ethernet switch failed, we would not lose communication to the entire payload.

We generally downsample and use jpeg compression on images to reduce the file size from ~20 MB to ~15 kB. Using only jpeg compression without downsampling reduces images to about 200 kB. We download a full sky view (images from each camera) every 2 min with the full resolution 200-kB images, when we can access either the high-gain TDRSS link or the Iridium link.

### 3.6. Ground Software

We developed custom ground software to display telemetry information. The software uses files prepared by the communicator, which includes custom image metadata. The communicator packetizes each file in chunks of 1,000 bytes. We developed this format to simultaneously use multiple communication channels with distinct communication protocols and track missing or incomplete files with the ground-side software.

When the communicator prepares data for downlink, it includes metadata with the packetized chunks indicating the communication channel and the packet number, along with the total packet numbers. This allows the ground-side software to track missing packets and aggregate packetized data back into complete files.

**Figure 7.**
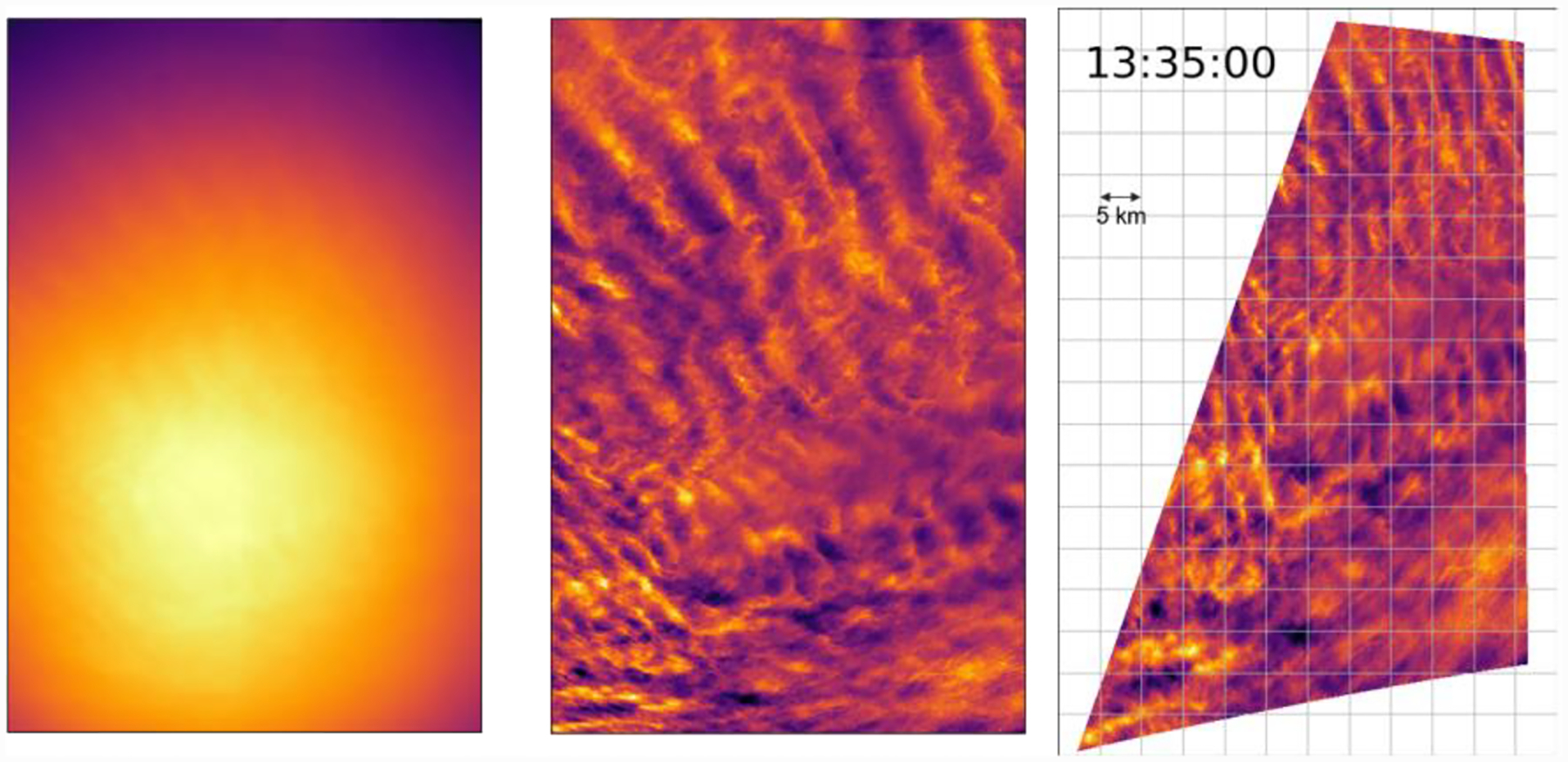
From left to right: raw false-colored image of one of the wide-field cameras containing PMCs; the same image after flat-fielding; and the same image after flat-fielding and projection based on the pointing solution. Up orientation in the first two images corresponds to the zenith, and up orientation in the projected image corresponds to the longitude of the Sun. We projected the image looking from below.

The communicator sends periodic housekeeping data contained in 256-byte packets, along the reliable Iridium short-burst data link. It also periodically intersperses the high-rate packetized data with housekeeping data. We wrote ground-side software to display these updates in a quickly readable manner for the ground monitoring team. We also wrote graphical user interfaces (GUIs) to display incoming files with the number of packets expected and received, as well as an image viewing GUI that includes image metadata (Figure 7).

We also wrote software to facilitate commanding the payload. It translates human-readable commands into a compact form and facilitates switching command uplink channels. The communicator also includes command verification that it sends down along with housekeeping. The command software tracks the command verifications and displays sent commands and whether the verification has been received.

Finally, we developed a GUI to display image files received by the ground computer. In addition to convenient image displaying features, this GUI displayed image metadata and generated commands to request a section of a specific image file. The GUI of this software is shown in Figure 7.

### 3.7. Flight Performance

The telemetry system met expectations during flight. We sent commands in real time in response to the changing sky conditions; we observed PMCs even in low-quality downlinked images, as shown in Figure 7; and we used the communication channels to run several live tests including using stars to dial in our focus settings with focus sweeps and measuring the sky brightness as a function of Sun angle.

We maintained communication with the payload over the LOS link from launch for 20 hr until 9 July 03:38 UTC. We dropped 1% of the downlinked packets over the LOS link during this time, but we did not encounter any periods of the link dropping completely. Iridium Pilot dropped 0.1% of the packets when we had connection. While we maintained Pilot connection for the vast majority of flight, we did encounter occasional Pilot outages. These outages typically lasted less than 30 min, but we did have two Pilot outages that lasted over an hour. TDRSS generally performed as well as Iridium Pilot. However, we encountered issues with our ground-side computers and periodically needed to restart the software. Our ability to debug these issues was limited since the ground-side computers were located in Texas, while our ground team stayed in Sweden for the duration of flight. Furthermore, a network issue affecting the Columbia Scientific Ballooning Facility that housed our computers cut off access to TDRSS communication. As a result of these issues, we have less reliable quantitative measurements of the TDRSS performance. Qualitatively, TDRSS worked well when we did not encounter software or networking errors.

**Figure 8.**
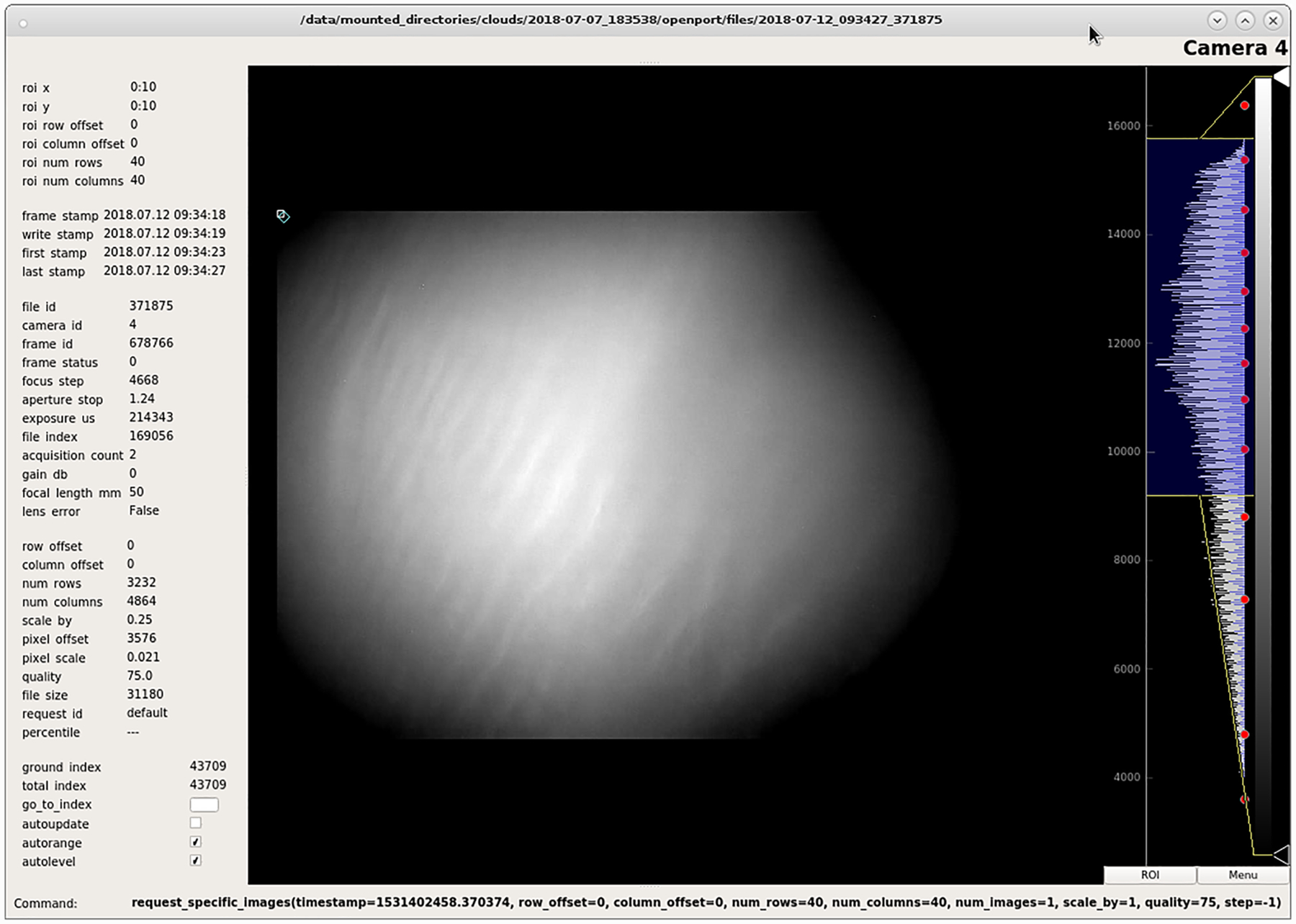
Screenshot of ground-based custom GUI taken during flight monitoring, displaying a downlinked image containing PMCs. The dynamic range has been stretched within the GUI software to emphasize the PMCs, visible as bright stripes oriented diagonally within the image.

During flight, we downlinked over 37,000 files over the LOS link, 245,000 files over Iridium Pilot and 257,000 files over TDRSS. Of these 540,000 files, about a third were compressed images. We did not need to use the compressed images for analysis, but they provided a backup option in case we had lost the payload Figure 8.

As expected, both TDRSS and Iridium Pilot occasionally lost communication, although not simultaneously. During the outages, the PMC Turbo subsystems continued to operate autonomously, including continuous data collection.

### 3.8. Comparison of Software and Computer Hardware With EBEX and BLAST

While the PMC Turbo cameras are similar to the star cameras developed for the EBEX and the Balloon-Borne Large-Aperture Submillimeter Telescope—the Next Generation (BLAST-TNG) (Galitzki et al., 2014; The EBEX Collaboration, Aboobaker, Ade, Araujo, Aubin, Baccigalupi, Bao, Chapman, Didier, Dobbs, Grainger, et al., 2018), the requirements from the software were very different. The function of the EBEX and BLAST cameras were to detect *stars*, and their primary role was in the experiments’ attitude control system, which included other inputs, most prominently gyroscopes (The EBEX Collaboration, Aboobaker, Ade, Araujo, Aubin, Baccigalupi, Bao, Chapman, Didier, Dobbs, Grainger, et al., 2018). With EBEX, for example, star camera software was designed to determine payload attitude with less than 2″ in az and el. In contrast, the PMC Turbo camera software has no payload attitude functions. When we want to know the pointing, we locate stars in our images and use Astrometry.net software to compare the star locations to a catalogue. However, such functionality was not required during flight. The camera operates at much higher cadence, and software functions are primarily image storage and preparing data for downlink.

EBEX and BLAST had more complex sky scanning patterns, instrument tuning demands, and observation scheduling compared to PMC Turbo. They implemented arrays of sensors operating at cryogenic temperatures that required relatively elaborate readout electronics. As a consequence, both payloads had dedicated flight control computers and computer-like readout boards in addition to their two camera computers. In contrast, with PMC Turbo, all software programs, including flight control, basic task scheduling, and other functions, run on the “camera” computers. The lidar included a dedicated computer and its own software, but like the pressure vessels, the main PMC Turbo software only needed to relay commands and diagnostics for this instrument.

The PMC Turbo model of distributed software and computer hardware will work for instrumentalists who want to develop redundant software for many independent instruments. For more complex instruments requiring coordination of more complex tasks, EBEX and BLAST-TNG would provide a better instrument blueprint.

## 4. Preliminary Results

PMC Turbo captured images of dynamic phenomena including GW breaking events, turbulent Kelvin-Helmholtz instabilities (present in Figures 5 and 7), mesospheric bores, self-acceleration events, and vortex rings (present in Figure 6). We are currently studying these events and comparing them to numerical simulations. We have published an overview paper (Fritts et al., 2019), describing GWs, associated instabilities, and the larger atmospheric context of our observations. We have also published a study of two successive mesospheric bores imaged by PMC Turbo (Fritts et al., 2020). The PMC Turbo hardware enabled analysis on an area of ~75 × 150 km over ~2 hr while also resolving the bore and instability features with scales down to ~100 m. This allowed for improved resolution of bore dynamics relative to ground-based observations.

We have written a study which utilized the imaging capabilities of the PMC Turbo instrument to quantify GW wavelength, propagation direction, phase speed, and mean energy dissipation rate (Gravity Wave Breaking and Vortex Ring Formation Observed by PMC Turbo, May 2020). Finally, we have identified Kelvin-Helmholtz instabilities and studied the small-scale structures of these phenomena. Our imaging capabilities enable resolution of small-scale dynamics which help constrain and construct models of GW-induced Kelvin-Helmholtz instabilities, and we are preparing observation and modeling companion papers covering these phenomena.

The lidar mounted on PMC Turbo observed bright and rapidly changing PMC layers and temperature perturbations derived from the Rayleigh backscatter data collected. Data from the lidar gave additional insight into the interpretation of specific turbulence events observed with the cameras (Fritts et al., 2019).

## 5. Summary

PMC Turbo is a balloon-borne instrument which imaged PMCs during a 6-day flight from Northern Sweden to Northern Canada in 2018. The instrument consists of a gondola hosting seven pressure vessels each of which contains a camera and a computer which controls image acquisition, communication, and system monitoring. The gondola also hosts a Rayleigh lidar, telemetry hardware, and a solar power system. Of the seven cameras, four use 50-mm lenses, and three use 135-mm lenses to obtain both wide and narrow FOVs. The full FOV stretches some 200 km × 100 km on the plane of the PMCs with a resolution of 8 m/pixel for the wide cameras and 3 m/pixel for the narrow cameras. The cameras captured 4 million images of PMCs during the 2018 flight.

We developed software optimized for recording PMC images and for controlling data collection. The software compresses and stores images, controls the camera hardware including focus and aperture, and communicates with other pressure vessels and gondola instruments. Our flight control software communicates with the ground, and we developed both the software and the network of instruments with a distributed control structure for communication resilience. This software will be distributed to the community via the PyHC SkyWinder package.

The software and network of PMC Turbo could provide a model for other instruments with a large data volume and detectors that do not require extensive fine tuning. Our camera hardware is similar to ground-based PMC experiments. However, the development of a balloon-borne platform to host the cameras allowed us to record a much larger amount of high-resolution data.

## Figures and Tables

**Table 1:** Comparison of Field of View and Pixel Area on the Plane of PMCs Between Instruments Imaging PMCs

Instrument	Field of view	Pixel area on the PMC plane
PMC Turbo	27° × 40° (wide) 10° × 15° (narrow)	8.2 m increasing with off-zenith angle (wide) 3.0 m (narrow)
EBEX	4.0° × 2.6°	2.5 m
CIPS	80° × 120°	1 km × 2 km (rectangular)
Ground-based cameras	130° × 85° (wide) 9.5° × 6.3° (narrow)	10–20 m (Baumgarten & Fritts, 2014)
SONC	110° × 82°	~30 to ~3,000 m

## Key Points:

- PMC Turbo, a balloon-borne platform hosting seven cameras and a Rayleigh lidar, launched in July 2018
- We describe the camera hardware and image capture methods used during the mission
- We describe the design and performance of the flight control hardware and software
